# Risk stratification and clinical course of hepatitis B virus reactivation in rheumatoid arthritis patients with resolved infection: final report of a multicenter prospective observational study at Japanese Red Cross Hospital

**DOI:** 10.1186/s13075-019-2053-1

**Published:** 2019-11-28

**Authors:** Wataru Fukuda, Tadamasa Hanyu, Masaki Katayama, Shinichi Mizuki, Akitomo Okada, Masayuki Miyata, Yuichi Handa, Masatoshi Hayashi, Yoshinobu Koyama, Kaoru Arii, Toshiyuki Kitaori, Hiroyuki Hagiyama, Yoshinori Urushidani, Takahito Yamasaki, Yoshihiko Ikeno, Takeshi Suzuki, Atsushi Omoto, Toshifumi Sugitani, Satoshi Morita, Shigeko Inokuma

**Affiliations:** 1 Center for Rheumatic Disease, Japanese Red Cross Kyoto Daiichi Hospital, 15-749 Honmachi, Higashiyama-ku, Kyoto City, Kyoto 605-0981 Japan; 2 Department of Rheumatology, Nagaoka Red Cross Hospital, 2-297-1 Senshu, Nagaoka-shi, Niigata 940-2085 Japan; 3 Department of Rheumatology, Osaka Red Cross Hospital, 5-30 Fudegasaki-cho, Tennoji-ku, Osaka city, Osaka 543-8555 Japan; 4 The Center for Rheumatic Diseases, Matsuyama Red Cross Hospital, 1 Bunkyo-cho, Matsuyama city, Ehime 790-8524 Japan; 5 Department of Rheumatology, Japanese Red Cross Nagasaki Genbaku Hospital, 3-15 Mori-machi, Nagasaki city, Nagasaki 852-8511 Japan; 6 Department of Internal Medicine, Japanese Red Cross Fukushima Hospital, 7-7 Yashima-cho, Fukushima city, Fukushima 960-8530 Japan; 7 Department of Rheumatology, Saitama Red Cross Hospital, 1-5 Shintoshin, Chuo-ku, Saitama city, Saitama 330-8553 Japan; 8 Department of Orthopedic Surgery and Rheumatology, Nagano Red Cross Hospital, 5-22-1 Wakasato, Nagano city, Nagano 380-8582 Japan; 9 Department of Rheumatology, Japanese Red Cross Okayama Hospital, 2-1-1 Aoe, Kita-ku, Okayama city, Okayama 700-8607 Japan; 10 Department of Internal Medicine, Japanese Red Cross Kochi Hospital, 1-4-63-11 Hadaminamimachi, Kochi city, Kochi 780-8562 Japan; 11 Department of Orthopedic Surgery, Japanese Red Cross Fukui Hospital, 2-4-1 Tsukimi, Fukui city, Fukui 918-8501 Japan; 12 Department of Rheumatology, Yokohama City Minato Red Cross Hospital, 3-12-1 Shinyamashita, Naka-ku, Yokohama city, Kanagawa 231-8682 Japan; 13 Department of Rheumatology, Matsue Red Cross Hospital, 200 Horomachi, Matsue city, Shimane 690-8506 Japan; 14 Department of Orthopedic Surgery, Tanabe Chuo Hospital, 6-1-6 Tanabechuo, Kyotanabe city, Kyoto 610-0334 Japan; 15 Department of Rheumatology, Nasu Red Cross Hospital, 1801-4 Nakadawara, Otawara city, Tochigi 324-0062 Japan; 16 Division of Allergy and Rheumatology, Japanese Red Cross Medical Center, 4-1-22 Hiroo, Shibuya-ku, Tokyo 150-8935 Japan; 17 Department of Biomedical Statistics and Bioinformatics, Kyoto University Graduate School of Medicine, Yoshidakonoe-cho, Sakyo-ku, Kyoto City, Kyoto 606-8501 Japan; 18 Department of Allergy and Rheumatology, Chiba Central Medical Center, 1835-1 Kasori-cho, Wakaba-ku, Chiba city, Chiba 264-0017 Japan

**Keywords:** Hepatitis B virus, Rheumatoid arthritis, Reactivation, Nucleic acid analog, Prognosis

## Abstract

**Background:**

The prophylaxis for hepatitis B virus (HBV) reactivation assumes that hepatic injury after reactivation is often rapidly progressive and can evoke fulminant hepatitis. The incidence and prognosis of reactivation in patients with rheumatoid arthritis (RA) may be different from those receiving organ transplantation and cancer chemotherapy. This study aimed to investigate the incidence, risk factors, and clinical course of HBV reactivation and develop a scoring system for risk stratification in RA patients with resolved infection.

**Methods:**

HBV DNA was measured using real-time polymerase chain reaction, and patient data were collected for 4 years in RA patients with resolved HBV infection who were treated with steroids or synthetic or biologic immunosuppressive drugs.

**Results:**

Among 1127 patients, HBV DNA was detected in 57 patients (1.65/100 person-years); none of the reactivated patients exhibited worsening of hepatic function. Multivariate logistical analysis revealed that age > 70 years and HB core antibody (HBcAb) positivity alone were independent risk factors for HBV reactivation. HBV DNA ≥ 2.1 log copies/mL was observed in 15 patients (0.43/100 person-years); seven patients were treated with nucleic acid analogs (NAAs), whereas the remaining eight were observed without treatment. Among reactivated cases, 15 cases changed to HBV DNA-negative status spontaneously, whereas 24 cases remained HBV DNA positive < 2.1 log copies/mL during the observation period. We designed the following scoring system: HBV reactivation risk score = 1 × (age > 70 years) + 2 × (HBcAb positivity alone) + 1 × (treatment other than methotrexate monotherapy). This revealed that patients with the highest score had an odds ratio of 13.01 for HBV reactivation, compared to those with the lowest score.

**Conclusions:**

Rapid progression and poor outcomes after HBV reactivation were not frequent in RA patients with resolved infection. Our new risk scoring system might be useful for screening and optimization of prophylactic treatment by distinguishing patients with significantly lower reactivation risk.

## Background

Hepatitis B virus (HBV) reactivation due to immunosuppressive therapy is an important therapeutic complication of rheumatic diseases. HBV reactivation is less frequent in patients with resolved HBV infection than in HBV carriers or those with chronic HBV hepatitis; however, the number of patients is several times more in the resolved infection group than in the chronic infection group. In addition, HBV reactivation was reported to be less frequent in patients with rheumatoid arthritis (RA) than those receiving cancer chemotherapy and transplantation [1]; however, the treatment period is longer for RA than that for chemotherapy and transplantation, requiring long-term monitoring for HBV reactivation. Therefore, HBV reactivation in RA patients with resolved HBV infection is an important issue for not only medical but also social and economic implications.

We previously conducted a multicenter prospective observational study on HBV reactivation in patients with RA and resolved HBV infection to determine the incidence and risk factors of HBV reactivation during a 2-year observation period and reported that the incidence of reactivation was low at 1.93/100 person-years but that the therapeutic risk should not to be neglected. Furthermore, we found that older age (≥ 69 years old) and low antibody titer against hepatitis B virus surface (HBs) antigen (HBsAg) were risk factors for HBV reactivation and that the risk persisted for a long time period after the initiation of immunosuppressive treatment [2].

HBV reactivation by immunosuppressive therapy is generally considered a rapidly progressive condition with poor prognosis that often leads to fulminant hepatitis [3, 4]. Based on this recognition, several guidelines and recommendations for the prevention of HBV reactivation during immunosuppressive treatment have been developed in Japan [5–7] and other regions [8–10]. Conversely, the risk of HBV reactivation is considered to differ between patients undergoing organ transplantation or cancer chemotherapy and those treated for rheumatic diseases [1]. Several studies also reported that the subsequent course and prognosis depended on the underlying cause of HBV reactivation [11].

We herein present the final report of our 4-year observational study that used multivariate analyses to determine the incidence and risk factors of HBV reactivation in patients with RA and resolved HBV infection. We also investigated the clinical course and patient outcomes after HBV reactivation in RA patients based on the longest 4 years of observation.

## Methods

This multicenter, observational, prospective study between 2013 and 2016 was conducted by a study group comprising rheumatologists from 16 Japanese Red Cross hospitals.

### Subjects

Patients eligible for enrollment were those over 18 years of age who were diagnosed with RA attending a clinic for rheumatic diseases in one of the 16 Japanese Red Cross hospitals in Japan. Patients treated with corticosteroids (≥ 5 mg prednisolone or its equivalent dose); immunosuppressive synthetic disease-modifying anti-rheumatic drugs (DMARDs), namely methotrexate, leflunomide, tacrolimus, and mizoribine or their equivalents; and/or biologic DMARDs, namely infliximab, etanercept, adalimumab, tocilizumab, abatacept, golimumab, certolizumab pegol, and/or tofacitinib, were tested for HBsAg, antibody against HBsAg (HBsAb), and antibody against HBV core antibody (HBcAb) using chemiluminescent immunoassays. Patients with negative HBsAg and positive HBsAb and/or HBcAb were enrolled. All subjects were HBV DNA negative at entry; this was confirmed by real-time polymerase chain reaction (RT-PCR).

### Registration

All data of the enrolled patients were recorded anonymously and sent as password-protected digital information to the Japanese Red Cross Kyoto Daiichi Hospital Center for Rheumatic Disease. The initial data collection was conducted from February 2013 to October 2015 and included the following information: basic patient characteristics such as age, sex, and disease duration; data related to hepatitis such as HBsAg, HBsAb, HBcAb titers, HBV DNA measured by RT-PCR, and aspartate and alanine transaminase levels within the last 3 months; immunological data such as blood lymphocyte count and serum immunoglobulin G levels; parameters related to disease activity such as tender and swollen joints, global visual analog scale score, Disease Activity Score 28 (DAS28) [12], C-reactive protein level, and erythrocyte sedimentation rate; and information on medications such as dose of steroids and methotrexate and status on the use of biologic DMARDs or other immunosuppressants. After the second year of observation, serial quantification of HBV DNA measured by RT-PCR, which was usually evaluated every 3 months according to the Japanese guideline [6], immunological data, parameters related to disease activity, and medication information were recorded.

### Primary and secondary endpoints

We defined HBV reactivation as a positive conversion of HBV DNA measured by RT-PCR and included cases with positivity < 2.1 log copies/mL, positivity with unquantifiable HBV DNA and abbreviated as PUHD. We consulted hepatologists regarding the guidelines [6–8] for cases with HBV DNA positivity ≥ 2.1 log copies/mL, positivity with quantifiable HBV DNA and abbreviated as PQHD, and administered nucleic acid analogs (NAAs) if necessary. The primary endpoint of this study was the frequency of HBV reactivation and PQHD in HBsAg-negative and HBsAb-positive and/or HBcAb-positive patients with RA. We also examined risk factors of HBV reactivation and analyzed the clinical and serological course after HBV reactivation as secondary endpoints. Because the result of annual observation of HBV DNA adopted a larger value among serial measurement, only those patients whose HBV DNA status was negative at all time points in the subsequent year were considered to have become HBV DNA negative in the annual observation.

### Development of risk scoring system for HBV reactivation in RA patients with resolved HBV infection

In the current study, we developed a scoring system for patient stratification based on HBV reactivation risk using risk factors identified by multivariate logistical regression analysis. Cutoff values were determined by receiver operating characteristic (ROC) analysis for continuous variables, and weighting was taken into consideration based on the strength of influence of each variable. Finally, we investigated the predictive ability of the reactivation risk score in the study cohort.

### Statistical analysis

Demographic factors and the primary endpoint were descriptively summarized. The predictive model was developed in terms of statistical significance of risk factors (*p* < 0.05) and the ease of clinical interpretation, in addition to C statistics with 95% confidence interval (CI). The model was developed based on the logistical model that used the HBV reactivation status as the dependent variable and the risk factors for HBV reactivation, such as age (< 65 vs ≥ 65 years), antibody titer, serum albumin, antibody positivity, and administration of drugs, as explanatory variables. Then, based on the odds ratios derived from the developed model, we assigned integer risk scores to each risk factor, such that the risk scores best reflect the point estimates of the odds ratio. Statistical analyses were performed using JMP version12.

### Ethics

In this study, we evaluated only data that were collected during the course of usual medical practice and substituted the agreement acquisition in the document with posting based on the Ethical Guidelines for Epidemiological Research [13]. The ethics committees of all contributing institutions approved the protocol for this study.

## Results

### The characteristics of the enrolled patients

Among 1429 cases registered in 2 years, 1127 cases (3520 person-years) were analyzed after excluding HBsAg-positive and dropout cases. Demographic data for each year are presented in Table 1. The average age was 68.2 years, and the duration of illness was 123.4 months at the time of registration; majority of the cohort included elderly patients with long-term illness. The average DAS28 was 3.18, indicating low disease activity. The percentages of patients receiving corticosteroids, biologics, and methotrexate at the time of enrolment were 42.2%, 26.7%, and 78.6%, respectively; however, the proportion of those receiving biologics increased despite a decline in the proportion of those receiving other treatments during the observation period. Among the biologics, majority of the patients received etanercept or tocilizumab. The data on HBsAb and HBcAb positivity are shown in Table 2. Although the patients positive only for HBsAb are sometimes considered negative for past HBV infection, the current study included 122 patients (10.8%) who did not have a clear vaccination history according to the Japanese guidelines [6].

**Table 1:** Demographic features of patients on each observation year

	Registration	1st year	2nd year	3rd year	4th year
Patients (*n*)	1127	1127	997	808	588
Age (years)					
Mean ± SD	68.2 ± 10.2	69.2 ± 11.1	70.0 ± 10.2	70.7 ± 9.8	71.0 ± 9.4
Median, IQR	69, 62–76	70, 63–77	71, 64–77	71, 65–78	71, 65–78
Sex, female (%)	799 (70.9)	799 (70.9)	714 (71.6)	595 (73.6)	441 (75.0)
Disease duration (months)					
Mean ± SD	123.4 ± 115.1	135.5 ± 115.5	147.7 ± 114.5	163.6 ± 114.1	177.3 ± 112.0
Median, IQR	93, 36–169	105, 48–181	117, 61–193	112, 80–207	147, 97–218
DAS28 score (average, SD)	3.18 ± 1.76	2.88 ± 1.58	2.92 ± 1.57	2.84 ± 1.52	2.83 ± 1.53
Change of prescription		456 (40.5)	360 (36.1)	282 (34.9)	191 (32.5)
Prednisolone					
Number of patients (%)	476 (42.2)	435 (38.6)	352 (35.3)	280 (34.7)	166 (28.2)
Average dose (mg/day)					
Mean ± SD	4.22 ± 2.54	4.25 ± 2.94	4.21 ± 2.77	4.13 ± 2.92	3.99 ± 2.83
≥ 5 mg, number (%)	243 (21.6)	204(18.1)	165 (16.5)	118 (14.6)	68 (11.5)
Biologic DMARDs					
Number of patients (%)	302 (26.7)	314(27.8)	282 (28.2)	244 (30.2)	199 (33.8)
Etanercept, number (%)	115 (10.2)	119 (10.6)	105 (10.5)	88 (10.9)	77 (13.1)
Infliximab, number (%)	34 (3.0)	30 (2.7)	24 (2.4)	22 (2.7)	12 (2.0)
Adalimumab, number (%)	35 (3.1)	28 (2.5)	25 (2.5)	18 (2.2)	14 (2.4)
Tocilizumab, number (%)	53 (4.7)	61 (5.4)	59 (5.9)	58 (7.2)	51 (8.7)
Abatacept, number (%)	28 (2.5)	34 (3.0)	40 (4.0)	34 (4.2)	31 (5.3)
Golimumab, number (%)	33 (2.9)	39 (3.5)	32 (3.2)	26 (3.2)	13 (2.2)
Others, number (%)	4 (0.4)	3 (0.3)	13 (1.3)	13 (1.6)	9 (1.5)
Methotrexate					
Number of patients (%)	886 (78.6)	835 (74.1)	731 (73.3)	581 (71.9)	414 (70.4)
Average dose (mg/week), mean ± SD	7.51 ± 3.83	7.49 ± 4.01	7.31 ± 3.96	7.20 ± 3.92	7.00 ± 3.84
Other immunosuppressive drugs					
Number of patients (%)	182 (16.1)	191 (16.9)	168 (16.8)	140 (17.3)	117 (19.8)
Tacrolimus, number (%)	139 (12.3)	160 (14.2)	137 (13.7)	114 (14.1)	96 (16.3)
Mizoribine, number (%)	32 (2.8)	32 (2.8)	29 (2.9)	24 (3.0)	19 (3.2)
Leflunomide, number (%)	6 (0.5)	6 (0.5)	7 (0.7)	6 (0.7)	6 (1.0)
Others, number (%)	5 (0.5)	3 (0.3)	2 (0.2)	3 (0.4)	3 (0.5)

*Abbreviations*: *SD* standard deviation, *IQR* interquartile range, *DAS28* Disease Activity Score 28

**Table 2:** Number of HBV-related antibodies in enrolled patients

	HBcAb negative	HBcAb positive	Total
HBsAb negative	0	218 (19.3%)	218
HBsAb positive	122 (10.8%)	787 (69.8%)	909
Total	122	1005	1127

*Abbreviations*: *HBs Ab* anti-hepatitis B virus surface antibody, *HBc Ab* anti-hepatitis B virus core antibody

### The incidence of HBV reactivation

As shown in Table 3, HBV reactivation, as defined by HBV DNA positivity, was observed in 57 cases (1.65/100 person-years) during the 4 years of observation, and PQHD was found in 15 patients (0.42/100 person-years). The risk of reactivation was present throughout the 4 years, even though the incidence of cases declined with the progression of observation. Median interval between a change of RA treatment and HBV reactivation was 33.5 months [IQR 12–56.75].

**Table 3:** Incidence of HBV reactivation in each observation year

	Year of observation	Number of cases	Sample size (person-years)	Incidence (/100 person-years)	Use of NAAs
Reactivated cases	1	23	1127	2.04	5
	2	17	977	1.74	3
	3	9	779	1.16	2
	4	8	565	1.42	1
	Total	57	3448	1.65	11
Patients with HBV DNA ≥ 2.1 log copies/mL	1	3	1127	0.27	4
	2	5	996	0.50	2
	3	5	802	0.62	1
	4	2	599	0.33	1
	Total	15	3524	0.43	8

*Abbreviations*: *HBV DNA* hepatitis B virus DNA, *NAA* nucleic acid analog

### Risk factors for HBV reactivation

**Fig. 1:**
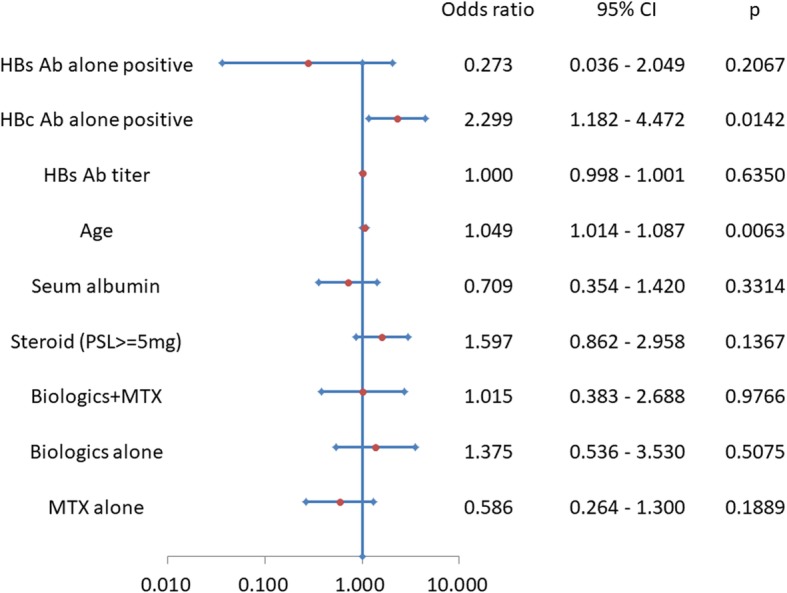
Odds ratios of clinical indicators for hepatitis B virus reactivation. Forest plot shows the odds ratios and 95% confidential intervals of clinical parameters calculated by multivariate logistical analysis for HBV reactivation in RA patients with resolved infection. Abbreviations: *HBs Ab* anti-hepatitis B virus surface antibody, *HBc Ab* anti-hepatitis B virus core antibody,* PSL *prednisolone, *MTX* methotrexate

The frequency of reactivation according to HBsAb/HBcAb positivity is shown in Table 4. Briefly, the highest frequency of 11.01% was observed in subjects who were positive only for HBcAb during 4 years of observation. In the current study, we performed multivariate logistical analysis using positivity for HBV-related antibodies, age, serum albumin, steroid administration, and administration of biologics and methotrexate, alone or in combination, as independent variables, which showed that age and a status of HBcAb positivity with HBsAb negativity were independent risk factors for HBV reactivation, as shown in Fig. 1. Although there were no differences in reactivation frequency among those treated with corticosteroids, biologics, and methotrexate, the odds ratio for reactivation(0.554 [95% CI 0.264–1.300]) was lower for patients treated with methotrexate not in combination with biologics compared to those treated with corticosteroid or biologics.

**Table 4:** The frequency of HBV reactivation for 4 years according to the positivity of HBs/HBc antibody in RA patients with resolved infection

	HBc Ab (+)/HBs Ab (+)	HBc Ab (+)/HBs Ab (−)	HBc Ab (−)/HBs Ab (+)	Total
Number	787	218	112	1127
Reactivated cases	32 (4.07%)	24 (11.01%)	1 (0.81%)	57 (5.05%)
Cases with HBV DNA ≥ 2.1 log copies/mL	6 (0.76%)	8 (3.67%)	1 (0.81%)	15 (1.33%)

*Abbreviations*: *HBV DNA* hepatitis B virus DNA, *HBs Ab* anti-hepatitis B virus surface antibody, *HBc Ab* anti-hepatitis B virus core antibody

### The outcome of HBV infection after reactivation

As shown in Table 5, among a total of 57 cases with HBV reactivation, observations of 24 cases were finished in 1 year. The observations for the second, third, and fourth years were possible in 17, 10, and 6 patients, respectively. Analysis of the outcomes at the final observation period of 57 cases with HBV reactivation revealed that 24 cases were PUHD (median observation period, 6.0 months; interquartile range [IQR] 1.5–21.3 months), 15 cases progressed to become PQHD (median of 9.0 [IQR 2.75–15.75] months from reactivation to PQHD), 15 patients became HBV DNA-negative (median of 10 [IQR 4–14.5] months from reactivation to negative conversion; median of 15.0 [IQR 9.5–18.5] months of observation after negative conversion), and 3 patients were treated with NAAs before becoming PQHD.

**Table 5:** Serological outcome of HBV-reactivated patients

Final observation	Number of patients	Serological outcome			
		PQHD	PUHD	HBV DNA negative	Others
1st year	24	6	15	0	3*
2nd year	17	6	3	8	0
3rd year	10	3	3	4	0
4th year	6	0	3	3	0
Total	57	15	24	15	3

*Patients with PUHD who received NAA treatment

*Abbreviations*: *HBV DNA* hepatitis B virus DNA, *PUHD* positivity with unquantifiable HBV DNA, *PQHD* positivity with quantifiable HBV DNA

### The course of HBV infection after PQHD (Fig. 2)

Clinical hepatic damage was not observed in any of the 15 patients with PQHD, and 7 of them received NAAs. Among the 9 cases that could be observed in the second year, HBV DNA was negative in 3 cases with and without NAA administration. Moreover, 3 out of 5 cases in the third year and 1 out of 2 cases in the fourth year were HBV DNA negative. The time interval of conversion from positivity to negativity were 1, 4, and 4 months for each of these patients, and the length of observation period after negative conversion were 10, 19, and 20 months, respectively.

**Fig. 2:**
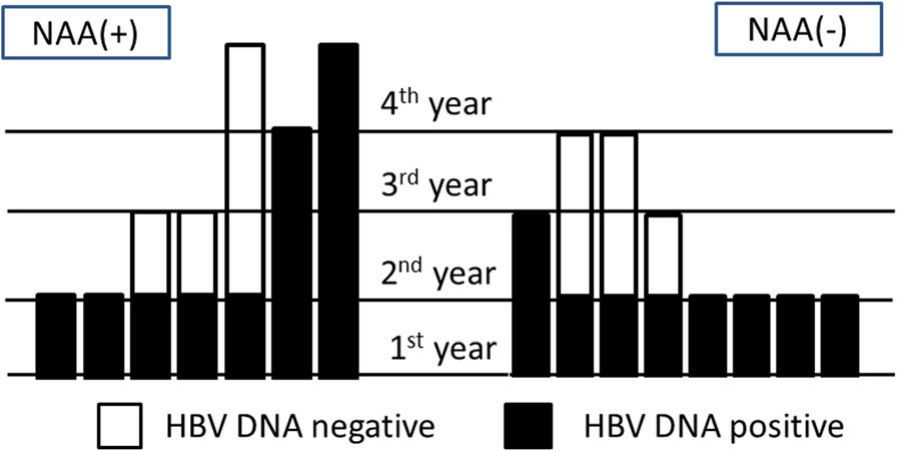
Serological outcomes after 15 patients became PQHD in the current study. Each of the 15 bars represents patients with PQHD, and the bar height represents the length of the observation period. The black portion indicates the year that HBV DNA was detected, and the white portion indicates the year that HBV DNA was negative. Of the 15 cases with PQHD, seven received NAA. HBV DNA status turned negative in 3 cases with and without NAA administration in the second year. Among five cases observed in the third and fourth years, the status of HBV infection did not change. Abbreviations: PQHD, positivity with quantifiable HBV DNA; HBV, hepatitis B virus; NAA, nucleic acid analog

### The development and validation of risk scoring for HBV reactivation in RA patients with resolved infection

As described above, HBcAb positivity alone and aging were significant risk factors for HBV reactivation in RA patients with resolved infection. Regarding age, the ROC analysis with HBV reactivation as a dependent variable revealed that the maximum “sensitivity − (1 − specificity)” was 0.2207 with an area under the curve (AUC) of 0.62914 when an age of 70 years was defined as the cutoff value. Furthermore, methotrexate monotherapy, albeit not a statistically significant independent variable, was the weakest risk factor compared to other therapeutic agents. Given that methotrexate is an anchor drug for RA, treatment other than methotrexate monotherapy, with prednisolone < 5 mg/day as an acceptable option in combination, was assessed for determining risk scoring. Among the three risk factors shown in Table 6, a risk scoring system was created with doubling the single HBcAb positivity, which had a particularly high odds ratio, as follows: risk score = 1 × (age > 70 years) + 2 × (HBcAb positivity alone) + 1 × (treatment other than methotrexate monotherapy).

**Table 6:** Odds ratio of risk factors used in scoring system for risk stratification of HBV reactivation in RA patients with resolved infection are shown

Risk factor	Number of patients	Odds ratio	95% CI
Age > 70 years	482	2.21	1.29–3.88
HBcAb (+) alone	218	3.28	1.88–5.66
Treatment other than MTX monotherapy	574	2.17	1.24–3.92

*Abbreviations*: *HBc Ab* anti-hepatitis B virus core antibody, *MTX* methotrexate, *CI* confidence interval

The risk score analysis was defined to determine the risk of HBV reactivation only in RA patients with resolved infection. Table 7 shows the number of patients and the odds of reactivation for each score (AUC = 0.694); our analysis revealed that the patient group with the full score had an odds ratio of 13.01 compared with the lowest-risk group.

**Table 7:** Odds ratio of HBV reactivation in each score point in scoring system for risk stratification of HBV reactivation in RA patients with resolved infection is shown

Risk score	Number of patients	Odds ratio	95% CI
0	283		
1	418	1.93	0.73–6.72
2	262	3.86	1.50–11.87
3	106	5.79	2.01–18.98
4	58	13.01	4.52–42.87

*Abbreviation*: *CI* confidence interval

## Discussion

In Japan, people with resolved HBV infection comprise approximately 23.1% of the total population, which is higher than that observed in Western countries [14]. Importantly, all patients with RA who receive immunosuppressive DMARDs including biologics are recommended for HBV screening and managed according to strict guidelines. Patients with negative HBsAg are screened for HBsAb and HBcAb, and those who are positive for either are monitored for HBV DNA by RT-PCR every 3 months. In patients with PQHD, prophylaxis with NAA is started immediately. Although the current study was conducted with the premise of compliance with this guideline, deviations from the guidelines were observed in some cases because the frequency of HBV DNA monitoring and the timing of NAA administration remain controversial.

In summary, our final analysis revealed that the incidence of reactivation (1.65/100 person-years) was lower at the end of the 4-year observation period compared with the 2-year incidence rate that we reported previously [2]. The frequency of HBV reactivation in RA patients with resolved infection in the current study was comparable to that reported in a recent Japanese study, and the risk of HBV reactivation was significantly lower in RA than in other diseases [1, 2, 15]. New reactivation cases were also observed in the third and fourth observation years in the current study. Mochida et al. reported that the risk of HBV reactivation in patients with immunosuppressive drugs decreased significantly 6 months after treatment initiation [11]. Our results, albeit not contradicting their conclusion, revealed that the risk of reactivation persisted for a long time after the start of immunosuppressive treatment for RA. Since the risk of HBV reactivation in RA patients with resolved infection is obviously lower than that in patients undergoing organ transplantation or cancer chemotherapy and persists for a long time, the management for HBV reactivation in RA patients should be considered separately from that in patients requiring HBV reactivation management for other causes.

Our multivariate logistical analysis for risk factors based on the data collected over the 4 years of observation revealed that aging and HBcAb positivity in patients with HBsAb negativity were significant risk factors. Among the therapeutic drugs for RA, the odds ratio for methotrexate as a risk factor was lower than those for other drugs, although a statistically significant difference was not found; this finding was in agreement with the results of the univariate analysis in our previous report [2].

The evaluation of the serological outcomes of 57 patients with HBV reactivation over a maximum period of 4 years revealed that 15 cases (26.3%) progressed to PQHD, whereas in the other 15 cases, HBV DNA-negative status spontaneously changed. It is uncertain whether HBV infection will be progressive or not after HBV reactivation. Even if it does occur, it does not always progress rapidly. The observation of patients with PQHD showed that NAA can prevent hepatitis after HBV reactivation regardless of whether HBV DNA turns negative or not. Because the status of three patients who did not receive NAA spontaneously became HBV DNA negative, we should not consider that PQHD always implies progressive infection. The guidelines for the management of HBV reactivation are based on studies demonstrating that the course after HBV reactivation is often rapidly progressive, with a high incidence of fulminant hepatitis and poor prognosis; these studies included primarily patients undergoing organ transplantation and chemotherapy [1, 16]. Reports of fulminant hepatitis due to HBV reactivation in RA patients with resolved infection is limited to several case reports [17, 18], and no studies to date reported the incidence of fulminant hepatitis after reactivation. The incidence of hepatitis could not be determined in the current study since intervention was performed by prophylactic NAA administration. However, studies in rheumatic diseases suggest that, in addition to the low incidence of HBV reactivation, the clinical course after reactivation is not necessarily rapidly progressive, and the prognosis is not poor. Therefore, the criteria for prophylactic NAA administration and monitoring for HBV DNA should be reevaluated with consideration of stratified management based on risk factors that were mentioned previously.

One potential reason for the absence of cases with fulminant hepatitis among those with HBV reactivation is continuation of immunosuppressive therapy after reactivation, which was implemented in all cases according to the Japanese guidelines. Considering that hepatitis was reported to develop after reduction or discontinuation of immunosuppressants in several case reports [18, 19], the current results are consistent with the hypothesis that immune reconstruction due to treatment discontinuation might contribute to the development of hepatic injury.

We also created a scoring system by combining multiple risk factors of HBV reactivation with the aim to stratify RA patients with resolved infection according to risk of HBV reactivation. Chen et al. used conventional synthetic DMARDs alone as a low risk factor and HBcAb single positivity as a moderate risk factor for stratification of HBV reactivation risk [20]. The risk factors used in the current study were consistent with that study; however, age > 70 years was a new, important risk factor that was incorporated to derive the risk score in the current study. The number of patients in each score group (Table 6) reflects the status in Japan, which is likely to vary widely across regions based on differences in major HBV genotypes, incidence of HBV infection, frequency of each DMARD use for RA, and degree of societal aging. The risk of HBV reactivation can also vary across regions, and the risk factors and weighting are necessary to verify our results for each region. Despite these limitations, we found that the risk of reactivation in patients with a risk score of 0 or 1 was very low; in contrast, the odds ratio for reactivation in patients with risk scores of 3 and 4 points were 5.79 and 13.01, respectively, indicating high risk. Considering that the disease course after HBV reactivation in RA was not aggressive, as revealed in the current study, routine monitoring may be changed to simpler and less expensive method in low-risk patients. As a result, 63% of the patients enrolled in the current study could have avoided periodic HBV DNA measurements, which would have reduced the economic burden on the patients and the public considerably.

The current study has several limitations that should be acknowledged. First, considering the frequency of reactivation, the sample size and the study period were insufficient, and the risk assessment could not be performed for specific biologics. Second, this was an observational study, and 10 patients, comprising 17.5% of the reactivated cases, received prophylactic NAA treatment in accordance with the guidelines, and the incidence of hepatitis was unknown. The possibility that rapidly progressive hepatitis might occur with low frequency in these patients cannot be ruled out. Third, all subjects were Japanese, and differences in race and virus genotypes were not included in the analyses. Since rituximab is not used for RA treatment in Japan, the possibility remains that the frequency of HBV reactivation is lower in Japan than in other countries [21]. The scoring system for risk of HBV reactivation must be validated for each region, as described above.

## Conclusions

Albeit relatively low, the risk of reactivation in RA patients with resolved HBV infection during immunosuppressive treatment was sustained during treatment. Rapid progression of hepatic injury after HBV reactivation was not observed, and negative conversion was found in some patients after HBV reactivation following a natural course. HBV reactivation in patients with RA was not associated with a clearly poor prognosis. Our predictive risk scoring system for HBV reactivation might be useful for monitoring of HBV reactivation in RA patients.

## Data Availability

The datasets used and analyzed during the current study are available from the corresponding author on reasonable request.

## Abbreviations

AUC: Area under the curve
CI: Confidence interval
DAS28: Disease Activity Score 28
DMARD: Disease-modifying anti-rheumatic drug
HBcAb: Antibody against hepatitis B virus core antigen
HBs Ag: Hepatitis B virus surface antigen
HBsAb: Antibody against hepatitis B virus surface antigen
HBV: Hepatitis B virus
IQR: Interquartile range
NAA: Nucleic acid analog
PQHD: Positivity with quantifiable HBV DNA, HBV DNA positivity ≥ 2.1 log copies/mL
PUHD: Positivity with unquantifiable HBV DNA, HBV DNA positivity < 2.1 log copies/mL
RA: Rheumatoid arthritis
ROC: Receiver operating characteristic
RT-PCR: Real-time polymerase chain reaction
